# Swedish Public Railway Transportation Environments May Be Associated With High Exposure to Radiofrequency Electromagnetic Field (RF-EMF) Radiation

**DOI:** 10.7759/cureus.117957

**Published:** 2026-10-15

**Authors:** Lennart Hardell, Mona Nilsson

**Affiliations:** 1 The Environment and Cancer Research Foundation, Örebro, SWE; 2 Swedish Radiation Protection Foundation, Adelsö, SWE

**Keywords:** environmental exposure assessment, personal exposure, public transportation, radiofrequency electromagnetic fields, railway station, rf-emf exposure, subway, sweden

## Abstract

Background

The increasing use of wireless communication technologies has substantially changed the radiofrequency electromagnetic field (RF-EMF) environment. Public transportation systems represent complex RF-EMF exposure environments, but there is limited information regarding maximum environmental RF-EMF levels during routine travel across different transportation settings.

Objectives

This study aimed to characterize maximum environmental RF-EMF power density in Swedish transportation environments, including the Arlanda Express, Stockholm Central Railway Station, the Stockholm subway system, and intercity railway travel between Stockholm and Örebro.

Methods

Broadband RF-EMF measurements were performed using a Safe and Sound Pro II exposimeter for at least 2 minutes at each measured point.

Results

RF-EMF exposure showed substantial spatial and temporal variability across all investigated environments. The highest maximum power densities were consistently recorded at Stockholm Central Railway Station, where repeated measurements at ground level exceeded the instrument's upper measurement limit (>2,500,000 µW/m²). Subway measurements showed considerable variability, with localized maximum values exceeding 200,000 µW/m². Lower levels were generally observed during railway travel between stations, although high RF-EMF power density occurred on some railway sections. Exposure levels during travel varied according to location, network conditions, and transportation environment.

Conclusions

Public transportation environments, particularly major railway stations, can represent locations with high maximum power density levels. The measured levels were below current exposure limits recommended by the Federal Communications Commission (FCC) and International Commission on Non-Ionizing Radiation Protection (ICNIRP), which are based on protection against established thermal effects and do not incorporate non-thermal biological effects. However, the measured levels substantially exceeded the precautionary exposure levels recommended in the EUROPAEM EMF guideline 2016 for Wi-Fi exposure during daytime of 10 µW/m^2^, for nighttime 1 µW/m^2^, and for sensitive persons 0.1 µW/m^2 ^to protect against non-thermal effects. Future studies combining broadband measurements, frequency-selective analyses, and personal dosimetry are needed to better characterize individual exposure during modern daily travel.

## Introduction

Health risks associated with exposure to radiofrequency electromagnetic field (RF-EMF) radiation have been a concern for decades. More than 25 years ago, an epidemiological study reported an increased risk of brain tumors associated with mobile phone use [1,2]. Subsequent studies, including investigations by the same Swedish research group and findings from the international Interphone study, also reported an increased risk of glioma among the heaviest mobile phone users. In 2011, the International Agency for Research on Cancer (IARC) at WHO classified radiofrequency radiation in the frequency band 30 kHz to 300 GHz as a possible human carcinogen, Group 2B [3]. The IARC classification was published as Monograph Volume 102 in 2013 based on the 2011 working group. Several authors have argued that subsequent epidemiological, animal, and mechanistic evidence fulfill criteria for classification of RF-EMF exposure as a Group 1 human carcinogen, although IARC has not re-evaluated RF-EMF since its 2011 classification as Group 2B [4-7].

Other biological effects have also been associated with RF-EMF exposure, including effects on the nervous system, DNA damage, oxidative stress, and adverse effects on the reproductive system [8]. For persons living near mobile phone base stations, symptoms such as sleeping problems, fatigue, headache, dizziness, vertigo, memory and concentration problems, heart arrhythmia, and chest pressure have been reported [9,10]. These symptoms are included in microwave syndrome or radiofrequency sickness [11,12].

Increasing concerns have been raised about the health effects of RF-EMF radiation. The existing safety standards, developed by the International Commission on Non-Ionizing Radiation Protection (ICNIRP) and the U.S. Federal Communications Commission (FCC), consider established thermal effects, whereas a number of researchers have argued that evidence also exists for non-thermal biological effects [13-16]. With new research emerging, limitations of current exposure standards have become increasingly apparent. In particular, questions have been raised regarding whether current exposure assessment methods adequately characterize the complex spatial and temporal variability of RF-EMF exposure associated with modern wireless communication systems, including 5G technologies [16,17].

Although epidemiological and experimental studies have investigated potential biological effects of RF-EMF, accurate characterization of environmental exposure remains an additional prerequisite for interpreting health studies. Transportation environments are of particular interest because they combine numerous RF-EMF sources with large numbers of exposed individuals.

Thus, a high density of wireless devices used in enclosed spaces occupied by large numbers of passengers may contribute to elevated RF-EMF exposure levels in public transportation systems. Railway stations, trains, airport terminals, and underground subway systems contain multiple fixed RF sources. In addition, passengers using mobile phones, tablets, laptops, and wearable devices contribute to overall RF-EMF exposure [18]. Exposure conditions in these environments are influenced by several factors, including passenger density, signal propagation, train speed, building construction materials, and adaptive power control of mobile devices [18,19]. In underground systems, attenuation of radiofrequency signals may increase handset transmission power where network coverage is limited. Consequently, RF-EMF exposure may vary substantially depending on network infrastructure, passenger density, and train environment [18,19]. RF-EMF exposure in public transportation may exhibit substantial spatial and temporal variability.

Characterization of RF-EMF exposure in transportation environments has become increasingly relevant because commuting represents a regular component of daily life for a large proportion of the population. Personal exposimeter studies have shown that transportation environments contribute substantially to overall daily RF-EMF exposure, often exceeding exposure levels measured in residential settings [20,21]. Previous investigations have also shown considerable variability between transportation modes, with elevated environmental RF-EMF levels reported in railway stations, airport terminals, subway platforms, and other locations where large numbers of wireless devices operate simultaneously [21]. However, measurements performed during travel frequently show rapidly changing exposure patterns associated with train movement, changing network conditions, and fluctuations in passenger activity [21].

Few studies have examined maximum environmental RF-EMF levels during routine travel or compared exposure patterns across different components of the railway system, including airport rail links, long-distance trains, subway systems, and major transportation hubs. Such information is important for understanding the range and variability of environmental RF-EMF exposure during everyday travel and for improving exposure assessment in epidemiological and environmental health research [21].

Environmental measurements of RF-EMF do not provide information on absorbed dose or health effects. Nevertheless, systematic characterization of environmental exposure is an essential component of exposure science that may support epidemiological investigations on health effects.

## Materials and methods

The present study investigated environmental maximum RF-EMF power density in several public transportation environments in Sweden, including the Arlanda Express, Stockholm Central Railway Station, the Stockholm subway, and intercity railway travel between Stockholm and Örebro. Broadband measurements with the Safe and Sound Pro II exposimeter were used to characterize the spatial and temporal variation in maximum RF-EMF levels. The true response detection range is between 400 MHz and 7.2 GHz. It was calibrated by the manufacturer and has an accuracy of ±6 dB. Maximum RF-EMF power density levels were recorded each time. The maximum reading for the meter used was 2,500,000 µW/m^2^. Each measurement was performed for at least 2 minutes. The person performing the measurements did not use or wear any wireless device. It should be stressed that broadband measurements cannot identify individual frequency bands or distinguish contributions from 4G, 5G, Wi-Fi, onboard systems, or passenger devices. No standardized temporal protocol was used. Further, maximum short-duration measurements cannot establish cumulative exposure.

The specific objectives of the studies were to (i) quantify maximum RF-EMF power density in representative railway environments, (ii) compare exposure levels between stations and railway segments, and (iii) describe variations in environmental RF-EMF associated with different modes of public rail transportation.

Ethics

This was an environmental study that did not include any personal information. Thus, approval by the ethical committee was not necessary.

## Results

Arlanda Express to Stockholm Central Station

Measurements were made from Terminal 5 via Terminals 3 and 4 to Stockholm Central Railway Station. The journey takes 30 minutes. As shown in Table 1, maximum RF-EMF power density radiation varied from 41,900 to 474,000 µW/m^2^. The median maximum level was 137,000 µW/m^2^. The highest levels were recorded at the starting point at Arlanda and at the end at Stockholm Central Railway Station. In total, seven measurements were made, each lasting at least two minutes. About 50% of the passengers in the wagon used a mobile phone during the travel.

**Table 1:** Maximum RF-EMF power density (µW/m²) measured on the Arlanda Express between Arlanda Airport and Stockholm Central Railway Station on June 19, 2026. Measurements were performed between 14:10 and 14:40. Each measurement lasted at least two minutes. RF-EMF, radiofrequency electromagnetic field.

Measurement	Maximum RF-EMF (µW/m²)
1: Terminal 5 Arlanda	190,000
2	45,100
3	71,700
4	41,900
5	183,000
6	137,000
7: Stockholm Central Station	474,000

Stockholm Central Railway Station

RF radiation was measured three times, all in the afternoon, see Table 2. On the ground level, the highest radiation levels were found on the west side, which is in the direction of the trains. All measurements were >2,500,000 µW/m^2^, which is the highest possible reading for the exposimeter used. Thus, the actual radiation may have been even higher. Also, the east side against the main entrance at street level showed high radiation levels for all three measurements, ranging from 1,300,000 to >2,500,000 µW/m^2^, yielding a median maximum of 1,500,000 µW/m^2^.

**Table 2:** Maximum RF-EMF power density (µW/m²) measured at Stockholm Central Railway Station on three occasions. The date and time of each measurement are shown in the column headers. RF-EMF, radiofrequency electromagnetic field.

Measurement location	Direction	June 19, 2026 (16:00)	June 23, 2026 (15:00)	June 24, 2026 (14:30)
Ground level	North	342,000	59,000	250,000
	West	>2,500,000	>2,500,000	>2,500,000
	South	175,000	342,000	468,000
	East	1,300,000	1,500,000	>2,500,000
	Median	821,000	921,000	1,484,000
Lower level (subway)	North	37,400	58,500	26,800
	West	60,900	613,000	782,000
	South	1,090,000	183,000	214,000
	East	38,500	55,300	56,300
	Median	49,700	120,750	135,150

In the basement, (lower level) highest radiation was measured on the west side, with a median of 613,000 µW/m^2 ^and a range of 60,900 to 782,000 µW/m^2^. For all three measurements at the basement level, the range of the median maximum value was 49,700 to 135,150 µW/m^2^. 

Stockholm subway

Subway RF-EMF radiation levels were measured on June 23, 2026, on one subway line from Stockholm Central Railway Station to Fruängen, the end stop on that line. The results are displayed in Table 3. All measurements were made in the afternoon on the same day. In total, there were 12 measurements on that line, including the end stop. The measurement was made for at least 2 minutes each time. The results showed no clear geographical pattern, although higher levels tended to be found close to Stockholm city. Further, the wagon was more crowded during the first part of the trip, from 15:30 to 16:00, from the Central Railway Station to Fruängen.

**Table 3:** Maximum RF-EMF power density (µW/m²) measured during travel on the Stockholm subway between T-Centralen and Fruängen on June 23, 2026. RF-EMF, radiofrequency electromagnetic field.

Station	Outbound journey (15:30) maximum RF-EMF (µW/m²)	Return journey (18:50) maximum RF-EMF (µW/m²)
T-centralen-Gamla stan	51,400	86,400
Gamla stan-Slussen	254,000	176,000
Slussen-Mariatorget	61,800	419,000
Mariatorget-Zinkensdamm	36,800	313,000
Zinkensdamm-Hornstull	26,300	57,700
Hornstull-Liljeholmen	43,400	40,300
Liljeholmen-Midsommarkransen	83,200	32,300
Midsommarkransen-Telefonplan	116,000	24,400
Telefonplan-Hägerstensåsen	214,000	218,000
Hägerstensåsen-Västertorp	240,000	15,800
Västertorp-Fruängen	231,000	31,000
Fruängen platform	154,000	93,000

Measurements were taken during one outbound journey from T-centralen to Fruängen (departure: 15:30) and one return journey from Fruängen to T-centralen (departure: 18:50). Travel time was approximately 30 minutes in each direction. Values are maximum RF-EMF levels expressed as µW/m².

The median maximum RF-EMF radiation level from Stockholm Central Railway Station to Fruängen was 99,600 µW/m^2^, with a range of 26,300 to 254,000 µW/m^2^. The highest measured level was between Gamla stan (Old Town) and Slussen, which is in the central city area. For the return trip, the median maximum value was 72,050 µW/m^2^, with a range of 15,800 to 419,000 µW/m^2^. Also, for that trip, the highest maximum radiation was recorded in the Slussen region.

On the Fruängen platform, the results were 154,000 and 93,000 µW/m^2^, respectively, thus similar results on both trips.

Train travel Örebro-Stockholm-Örebro

Table 4 displays the results for railway travel on June 23, 2026, between Örebro and Stockholm Central Station. The median maximum RF-EMF radiation was 85,000 µW/m^2^, with a range of 8,610 to 1,030,000 µW/m^2^. The highest result was recorded at Arboga station, compared with lower readings during travel. On the platform at Örebro Central Station, 259,000 µW/m^2^ was recorded.

**Table 4:** Maximum RF-EMF power density (µW/m²) measured during railway travel from Örebro to Stockholm Central Railway Station, Sweden (SJ train 172, June 23, 2026). *On platform. **N.A. = not assessed; one measurement. RF-EMF, radiofrequency electromagnetic field.

Location/train segment	Number of measurements (n)	Median maximum RF-EMF (µW/m²)	Range of maximum RF-EMF (µW/m²)
Örebro Central Station*	1	259,000	N.A.**
Örebro Central Station	3	187,000	69,000-218,000
Örebro-Arboga	4	40,350	8,610-143,000
Arboga Station	1	1,030,000	N.A.**
Arboga-Köping	3	36,800	10,900-98,400
Köping Station	1	118,000	N.A.**
Köping-Västerås	6	74,900	8,930-275,000
Västerås Station	1	66,600	N.A.**
Västerås–Enköping	6	180,000	13,700-736,000
Enköping Station	1	400,000	N.A.**
Enköping-Bålsta	4	57,400	29,400-250,000
Bålsta Station	1	458,000	N.A.**
Bålsta-Sundbyberg	6	227,000	53,300-651,000
Sundbyberg Station	1	41,100	N.A.**
Sundbyberg-Stockholm Central	3	166,000	52,300-613,000
Stockholm Central Station	1	14,000	N.A.**

Similar results were obtained on the return travel from Stockholm Central Station to Örebro; see Table 5. The median maximum result was 67,800 µW/m^2^, with a range of 10,700 to 714,000 µW/m^2^. On the platform at Stockholm Central Railway Station, 16,500 µW/m^2^ was recorded (not in the table) compared with 52,300 µW/m^2^ on the platform at Örebro Central Station.

**Table 5:** Maximum RF-EMF power density (µW/m²) measured during railway travel from Stockholm Central Railway Station to Örebro, Sweden (SJ train 179, June 24, 2026). *N.A. = not assessed; one measurement. **On platform. RF-EMF, radiofrequency electromagnetic field.

Location/train segment	Number of measurements (n)	Median maximum RF-EMF (µW/m²)	Range of maximum RF-EMF (µW/m²)
Stockholm Central Station-Sundbyberg	6	66,650	24,400-183,000
Sundbyberg	1	104,000	N.A.*
Sundbyberg-Bålsta	6	115,000	102,000-714,000
Bålsta	1	30,000	N.A.*
Bålsta Station-Enköping	5	35,400	30,000-100,000
Enköping	1	78,700	N.A.*
Enköping-Västerås	6	50,000	20,700-123,000
Västerås	1	67,800	N.A.*
Västerås-Köping	5	64,700	39,600-88,000
Köping	1	52,300	N.A.*
Köping-Arboga	5	82,200	27,800-166,000
Arboga	1	25,800	N.A.*
Arboga-Örebro Central station	7	45,100	10,700-112,000
Örebro Central Station	1	114,000	N.A.*
Örebro Central Station**	1	52,300	N.A.*

Interestingly, the highest results were found both ways on June 23, 2026. The wagon was fully occupied during most trips, mostly by middle-aged or younger people. Further, most people used the mobile phone almost constantly, and nobody read a book or a newspaper.

## Discussion

Wireless RF-EMF radiation is now ubiquitous and difficult to avoid [21,22]. Environmental RF-EMF exposure has increased in many settings following the deployment of 5G networks, although measured levels vary considerably depending on local infrastructure and traffic [22-24]. Sources of environmental RF-EMF include mobile phones, laptops, base stations, modern vehicles, and public transportation systems.

We have previously reported high RF-EMF radiation levels at public places, such as Stockholm Central Railway Station. In a study with measurements in November 2015, the EME Spy 200 exposimeter was used. It is band-selective and covers 88 to 5,850 MHz. Based on 1,669 measurement points, the total maximum RF-EMF power density was 155,263 µW/m^2^ [25].

Our next investigation on RF-EMF radiation measurements at Stockholm Central Railway Station was in October 2023 [26]. The Narda broadband field meter NBM-550 equipped with the probe EF-1891 was used. The covered frequencies were 3 MHz to 18 GHz, covering previous generations of mobile communication and 5G. The recorded maximum RMS level on the ground floor was 68,452 µW/m² (one measurement), while basement measurements ranged from 599,207 to 3,637,191 µW/m² (six measurements). The ground floor result should be interpreted cautiously because only one measurement was performed.

Different exposimeters were used in the various studies (EME Spy 200, Narda NBM-550, and Safe and Sound II in the current study), so the results are not exactly comparable. It should be noted that RF-EMF levels at a given location may vary substantially between measurements taken at different times of the day and on different occasions. Certainly, the hypothesis that RF-EMF radiation levels are higher after the deployment of 5G should be further tested with frequency-selective, repeated measurements.

In a previous study, we measured very high RF-EMF radiation in the passenger cabin of an airplane with high-speed Wi-Fi [27]. The present measurements demonstrate that RF-EMF exposure during rail travel in the Stockholm region varies substantially. The highest exposure levels were recorded at Stockholm Central Railway Station, whereas lower levels were measured inside the Arlanda Express and the Stockholm subway. These findings indicate that railway stations may represent RF-EMF exposure "hotspots" compared with the journey itself.

On the Arlanda Express, peak RF-EMF levels varied by more than one order of magnitude, with the highest values recorded at Arlanda Airport and Stockholm Central Station. This pattern is consistent with the expectation that stations have a high density of active wireless communication systems and a large number of mobile phone users. During the journey, approximately half of the passengers in the wagon were observed using mobile phones. Personal devices may have contributed to measured RF-EMF levels, together with onboard wireless systems and signals from nearby base stations [21]. The generally lower exposure levels between stations may reflect fewer active transmitters in the immediate vicinity and lower passenger activity.

The highest RF-EMF levels of the entire study were measured at Stockholm Central Railway Station. On the ground level facing the railway tracks, the exposimeter repeatedly reached its upper detection limit (>2,500,000 µW/m²), indicating that the true maximum exposure exceeded the upper measurement range of the instrument (>2,500,000 µW/m²).

High RF-EMF radiation at the Stockholm Railway Station was measured already in October 2023 after the implementation of 5G [26]. Thus, our measurements demonstrate that Stockholm Central Railway Station is characterized by exceptionally high RF-EMF exposure. The consistently higher levels on the western side of the station than at the eastern entrance suggest that the railway platforms and their associated communication infrastructure constitute the dominant RF-EMF sources. In addition to fixed base station antennas, the large number of passengers using wireless devices is likely to contribute substantially to the observed exposure levels.

Measurements at the basement level of Stockholm Central Station yielded lower RF-EMF levels than those on the ground floor, although exposure remained higher than that measured during train travel. This differs from our results in 2023, which showed higher levels than those now measured [26]. This may be explained by different proximity to transmitting antennas and variations in passenger density. However, the marked spatial variation observed within the same station illustrates the importance of measurement location when characterizing RF-EMF exposure in complex public environments.

In the Stockholm subway, RF-EMF levels were generally lower than at Stockholm Central Station but were comparable to those measured on the Arlanda Express. Although no clear geographical trend was observed, the highest maximum values occurred around the central city area, particularly between Gamla stan and Slussen. These stations represent one of the busiest sections of the Stockholm metro network and are characterized by high passenger turnover and intensive mobile phone use, factors that may increase ambient RF-EMF levels. Passenger density was also higher during the outbound journey, which may partly explain the somewhat higher exposure compared with the return trip, although the highest individual maximum value was recorded during the return journey.

The considerable variability observed both within and between transport environments illustrates the dynamic nature of RF-EMF exposure during public transportation. Exposure is likely determined by multiple interacting factors, including passenger density, mobile phone activity, proximity to cellular base stations or distributed antenna systems, train and station design, and signal propagation characteristics within enclosed metallic structures. Since measurements were taken over relatively short periods, transient events such as simultaneous mobile phone transmissions may also have influenced individual maximum values.

From an epidemiological perspective, these findings indicate that railway stations, particularly major transport hubs, may contribute disproportionately to cumulative RF-EMF exposure among regular commuters. Time spent waiting on platforms may therefore account for a larger fraction of total exposure than the train journey itself. This observation highlights the importance of including transportation microenvironments in personal exposure assessment, as studies relying solely on residential or occupational exposure may underestimate total daily RF-EMF exposure.

The measured levels are below current international exposure limits, including those recommended by ICNIRP [13] and FCC [14,15]. They are based on acute adverse effects related to tissue heating. ICNIRP 2020 limits allow exposure as high as 10,000,000 µW/m^2^, measured as average level over 30 minutes. On the other hand, the levels recorded in this study are considerably higher than the precautionary levels recommended in the EUROPAEM EMF guidelines 2016, intended to protect against non-thermal effects and long-term exposure. The EUROPAEM guidelines recommend daytime precautionary levels of 10 µW/m² for Wi-Fi exposure, 1 µW/m² during nighttime, and 0.1 µW/m² for particularly sensitive individuals [28]. Note that the used instrument in our study measured broadband RF-EMF from multiple sources, not Wi-Fi specifically.

The study has several limitations. Measurements were performed on a limited number of occasions and during afternoon hours only, and therefore do not capture temporal variation associated with different times of day, weekdays, seasons, or fluctuations in passenger traffic. The exposimeter also reached its upper measurement limit at several locations within Stockholm Central Station, preventing determination of the actual maximum exposure. Limitations also include the use of short-duration maximum broadband measurements rather than time-averaged or personal exposure metrics, lack of standardized positioning and passenger-density measurements, and limited ability to attribute measured levels to specific sources such as 4G, 5G, Wi-Fi, or passenger devices. The manufacturer specifies an accuracy of ±6 dB for the Safe and Sound Pro II over the frequency range 400 MHz to 7.2 GHz. This represents a relatively large tolerance for quantitative power-density measurements.

Furthermore, information on the exact contribution from different RF sources, including mobile base stations, onboard communication systems, Wi-Fi, and passengers' personal devices, was not available. Future studies combining broadband measurements with frequency-selective analysis and repeated measurements under varying traffic conditions would provide a more comprehensive characterization of RF-EMF exposure in railway environments.

## Conclusions

Overall, the results in this study show that RF-EMF exposure during rail travel is highly heterogeneous, with the highest levels occurring at major railway stations rather than during travel between stations. These data contribute contemporary information on RF-EMF exposure in Swedish public transportation systems and may improve exposure assessment in epidemiological studies investigating potential health effects of RF-EMFs.
